# Resilience to climate change: from theory to practice through co-production of knowledge in Chile

**DOI:** 10.1007/s11625-016-0400-6

**Published:** 2016-10-03

**Authors:** Roxana Borquez, Paulina Aldunce, Carolina Adler

**Affiliations:** 1 Department of Geography, King’s College London, Room K4.10, Fourth Floor, Strand Campus, London, WC2R 2LS UK; 2 Center for Climate and Resilience Research (CR2), Blanco Encalada 2002, 4 Piso, DGF, Universidad de Chile, Santiago, Chile; 3 Department of Environmental Science and Resource Management, Faculty of Agricultural Science, University of Chile, Santa Rosa 11.315, La Pintana, Santiago, Chile; 4 Institute for Environmental Decisions and Transdisciplinarity Lab, D-USYS, ETH Zurich, CHN H 73.1, 16 Universitaetstrasse, 8092 Zurich, Switzerland

**Keywords:** Co-production, Resilience, Climate change, Transdisciplinarity, Drought, Chile

## Abstract

In theory, building resilience is touted as one way to deal with climate change impacts; however, in practice, there is a need to examine how contexts influence the capacity of building resilience. A participatory process was carried out through workshops in regions affected by drought in Chile in 2014. The aim was to explore how resilience theory can be better applied and articulated into practice vis-á-vis participatory approaches that enrich the research process through the incorporation of co-produced. The results show that there are more differences in responses by type of actor than between regions, where issues of national interest, such as ‘education-information’ and ‘preparedness’, are highlighted over others. However, historically relevant local topics emerged as differentiators: decentralisation, and political will. This reinforces why special attention must be given to the different understandings in knowledge co-production processes. This study provides evidence and lessons on the importance of incorporating processes of the co-production of knowledge as a means to better articulate and transfer abstract concepts, such as resilience theory, into practice.

## Introduction

Climate change poses a great challenge to society, with urgent responses needed that contribute to building resilience and developing adaptation (Aldunce et al. 2016). However, building resilience first requires an understanding for the ways, in which the term is understood and articulated by diverse actors in a given situation. In resilience frameworks, key elements have been described for building resilience in the context of a changing climate. These emphasise the importance of participatory processes and the co-production of knowledge, and the participation of stakeholders and decision makers, but with a little grounded validation. The purpose of this study was to address this gap, exploring how resilience theory can be applied and articulated into practice through the participatory approaches as a means to incorporate and ‘co-produce’ knowledge.

A number of arguments support the idea that expanding the participation of actors for the co-production of knowledge is valuable for sustaining resilience. For example, diversity in group composition is considered important, where participation from a diverse range of views or considerations for discussion is emphasised (Aldunce et al. 2016; Berkes 2007; Folke 2006; Gero et al. 2011). Others orient this participation as a process where giving opportunities to share knowledge and expertise are important, recognising that all knowledge are relevant (Armitage et al. 2008; O’Brien et al. 2010; Thomalla and Larsen 2010), or in providing space to meet different needs and enabling learning and changing actions (Wildavsky 1988).

Given the scale and complexity of building resilience to climate change, responses require active participation from governments, citizens, scientists, and private sectors. Nevertheless, several critiques have emerged with regard to the role that these different actors play in this participation, as for example, the role of scientists. Scientists have generated theoretical frameworks on many subjects related to climate change in order to understand and parameterise its effects, as well as people/community responses to climate change (Berrang-Ford et al. 2011; Djalante and Thomalla 2010). However, among scientists, it has accomplished little agreement on how adaptation or resilience is built, analysed, and applied in practice, incorporating different criteria and methodologies (Berrang-Ford et al. 2011; Vogel et al. 2007).

The science–society relationship has been often understood as mainly unidirectional, where academia provides research and knowledge in return for public funds (Gibbons 1999), where communities are viewed as subjects of inquiry without asking them about key issues that allow them as communities to adapt or to be resilient (Aldunce et al. 2014a; Vogel et al. 2007; Warburton and Martin 1999). Nonetheless, a growing number of scientists are looking towards “the opening up of knowledge systems”, enhancing relationships and collaboration between science and other actors, and orienting academic practice toward society (Cornell et al. 2013, p. 61).

On the whole, integrated research accounts for the complexity inherent in real-world problems (Miller et al. 2008; Cornell et al. 2013; Pohl et al. 2010), by integrating diverse knowledge systems in the process of co-producing knowledge. These knowledge systems should not only be understood as a collection of facts, but also as diverse ways, in which people understand, interpret, and give meaning to the world based on their experiences (Blaikie et al. 1997; Warburton and Martin 1999). In this paper, our goal is to complement knowledge on how to build resilience by demonstrating with case-based evidence how theory can be articulated into practice, how knowledge co-production can enrich the research process between context, regions, and actors, and how participant inputs can be explained/contextualised. The findings are based on the results of workshops on building resilience to climate change which took place in three regions in Chile during 2014.

This paper is organised into four sections. In ‘‘Introduction’’, we summarise key literature that has guided this research and contextualise the case study. ‘‘Resilience: moving from theory to practice through co-production of knpwledge’’ details the research design and methodology used. In ‘‘Context of application’’, we present the results and discuss how these results inter-relate to each of the communities where we conducted these workshops as well as present an overarching analysis of what these results mean in the context of bringing theory to practice. Finally, we conclude ‘‘Materials and methods’’ with reflections, lessons, suggestions, and challenges for moving forward on the topic.

## Resilience: moving from theory to practice through co-production of knowledge

The resilience perspective has influenced many disciplines and fields of research, including climate change (Maxwell 2009; Pelling 2011; Vogel et al. 2007; Walker and Salt 2006; Adler et al. 2015). However, there is no unique resilience framework, and its definition varies across different disciplines (Aldunce et al. 2015; Downes et al. 2013; Nelson et al. 2007). Literature on resilience specific to climate change includes a wide range of definitions and characteristics of resilience building (Aldunce et al. 2014a). According to Brandt et al. (2013), this diversity in conceptualisations is considered a weakness of the approach. However, we argue instead that this diversity is positive, because it gives flexibility which can be applied to different contexts and is part of the complexity inherent in the real world. Moreover, we believe that this diversity can be addressed through the transdisciplinary approaches that account for this complexity, because resilience cannot be reduced or simplified to one single perspective.

From a transdisciplinary perspective, moving from theory to practice through the co-production of knowledge involves the development of “empirical and practice-oriented knowledge than can help solve, mitigate or prevent life-world problems” (Pohl and Hirsch Hadorn 2007, p. 26). Thus, based on the conceptualization that “knowledge is a precondition for learning”, co-production of knowledge can be seen as a mechanism which enables learning, where five process dimensions play a role: “(1) knowledge gathering, (2) knowledge sharing, (3) knowledge integration, (4) knowledge interpretation, and (5) knowledge application” (Armitage et al. 2011, p. 998). Consequently, this type of research addresses socially relevant problems through “contextualised knowledge”, a concept coined by Gibbons (1999, C82) to explain the outcome of a mutual transformation between science and society. This includes reverse communication flows, where scientists seek socially robust knowledge, beyond scientific certainty, recognising that changes involve a complex interaction and feedback into socio-ecological systems over temporal and spatial scales (Gibbons 1999; Cornell et al. 2013).

In the context of our study, we applied a participatory methodology that included the delivery of information to participants. This information was the results of a systematic literature review on resilience to climate change for articles published between 2000 and 2012, where 15 determinants and 33 attributes were identified for resilience (for a detail description of determinants and attributes, see Aldunce et al. (2014b)), and factual data on drought in each region. This information was the starting point of the workshops. Thus, academic, social, private, and public actors jointly analysed that which aspects of the theory are relevant for their resilience building. Furthermore, scale and context were considered, recognising the context-specific uniqueness and mutual-influence between global and local scales (Olwig 2012).

## Context of application

Context is important for addressing the problem and the solutions proposed to address them, because cultural differences can influence perception, selection of options, interest, and meaning (Gibbons 1999; Wolf and Moser 2011). Thus, territorial aspect, multi-level governance, and climate drivers in the Chilean context were considered.

**Fig. 1:**
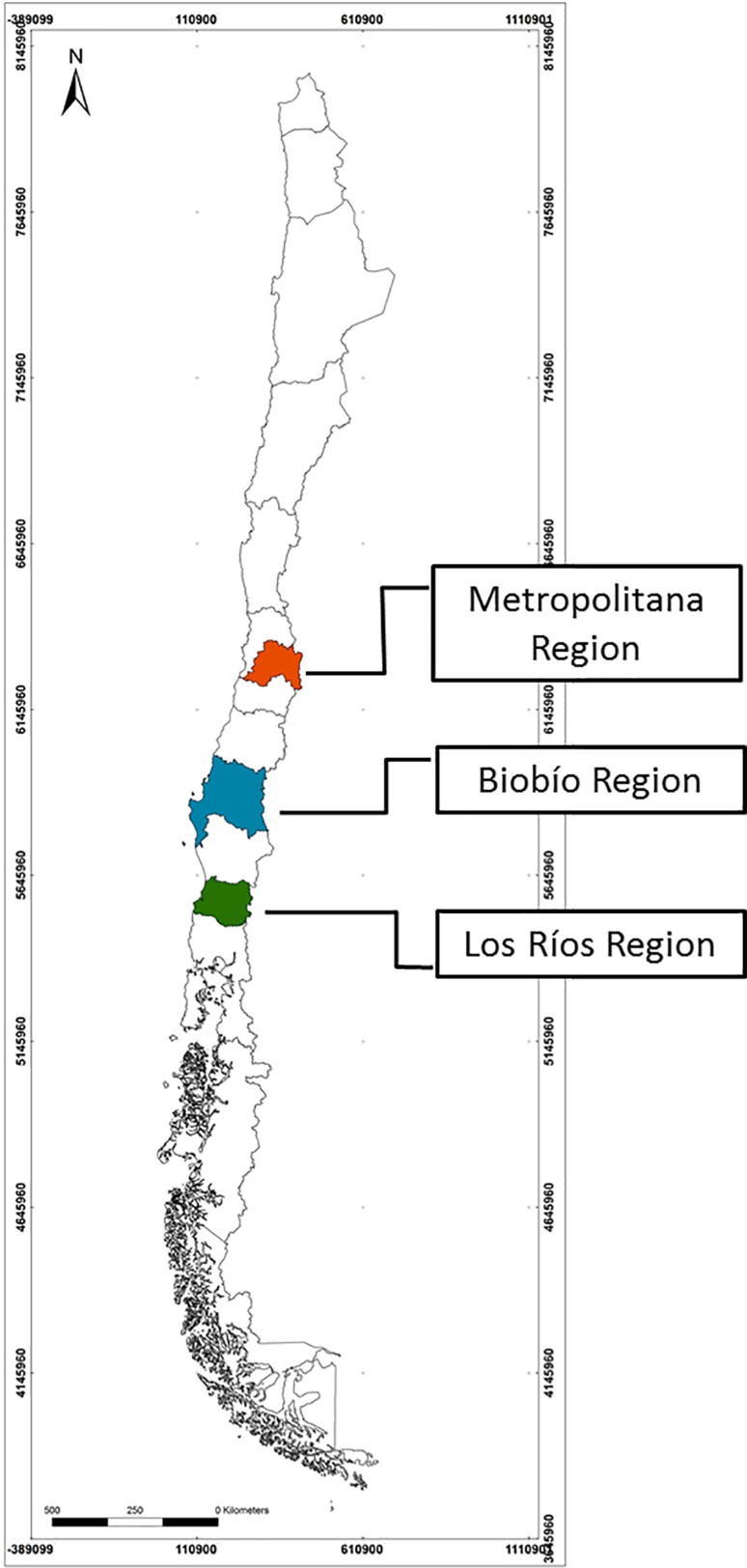
Geographical location of regions where workshops were developed

We carried out workshops in three Chilean regions: Los Ríos (*Región de Los Ríos*), Biobío (*Región del Biobío),* and Metropolitan (*Región Metropolitana de Santiago*). These three regions have distinct bio-physical, social, and economic conditions that can influence diverse responses to climate change (see Table 1). Figure 1 shows the geographical location of regions.

**Table 1:** Regional summary Sources: INE (2010), INE (2012), INE (2014), BCN (2016), Ilustre Municipalidad de Valdivia (2016)

Region	Climate	Average of annual precipitation (mm)	Population density (inhabitants/km^2^)	Main economic activities	Rural population (%)
Los Ríos	Temperate oceanic	1200–5500	21.8	Business	31.4
Biobío	Mediterranean	1200–2000	56.7	Forestry	16.3
Metropolitana	Mediterranean	356.2	469.3	Forestry agriculture	3.4

Chile has a diverse climate and topography, where precipitation deficit counts as one of the most important effects of climate change in this country (Meza et al. 2010). For these reasons, our study focused on drought as characterised by its slow onset, gradual impacts, cumulative effects, and conditional sensitivity to the context, in which it manifests. Therefore, it is important to highlight different effects that drought has on lifestyles, productive systems, and ecosystems due to the dependency to rainfall and/or water flows in the basis (freshwater, irrigation, energy production, and others) in different regions of Chile (Meza et al. 2010). According to Garreaud (2011), the projected changes in Chile between 2070 and 2100 (under A2 scenario—worst case—, using the PRECIS‐DGF model) show that precipitations on Los Ríos and Biobío Region would decline strongly (a reduction of 60–70 % from the climatic baseline 1960–1990), and in Metropolitan region, precipitations would decrease between 15 and 30 %.

## Materials and methods

Study phases and activities are shown in Table 2. The main framework of this study was based on the previous research developed by Aldunce et al. (2014b), where a systematic literature review of climate change resilience concept was performed for articles published between 2000 and 2012.^1^ As a starting point for input into our workshops, we used the identified 15 determinants and 33 attributes defined for resilience (Table 2).

**Table 2:** Phases and activities of study Source: drawn from example by (Singh et al. 2012) and (Singh et al. 2013b)

Phases	Period	Activities	Details
Preparatory	Jan–Dec 2013	Systematic literature review (Aldunce et al. 2014b)	Articles published on resilence to climate change, between 2000–2012. Scopus
	Jan–Apr 2014	Methodological design Objectives Principles and protocol Instruments Identification of target groups Selection of participants Facilitator Guide and training Material preparation	Collaboration of researchers from: Institute for environmental decision. ETH Zurich Stockholm resilience center Center for climate and resilience research, Chile
	May 2014	Invitation and confirmation of participants Sending invitationsvia email Calling to participants Remenbering invitation, place and time	289 invitations sent
Field work	Jun 2014	Workshops Valdivia, Los Ríos Region. June 3. Concepcion, Biobío Region. June 4. Santiago, Metropolitana Region. June 11.	61 participants
Analytical	Jun–Aug 2014	Analysis of instruments Thematic analysis (QSR Nvivo 10) Descriptive statistical of frequency	Individual questionnaire onResilience part A and B Individual questionnaire on attributes Wheel and transcription of discussion on attributes
Informative	Sep 2014	Seminar for returning results	Santiago: 76 participants. Via straming.: 21 participants (from Chile: Concepcion, Santiago, Valdivia; from other countries: Colombia, Brazil, Mexico)

Three data collection instruments were used during the workshops: two individual questionnaires and discussion groups.

Based on a project protocol, each workshop was designed in seven consecutive phases:

1. The initial questionnaire on resilience [first instrument part (a)] was completed by participants before the start of each workshop. This questionnaire sought to explore their level of knowledge of resilience.
2. Introduction (including workshop objectives, the study process, its strengths, limitations, and scope, who were the participants, why they were invited and why they were important in the process) and a presentation on drought in Chile, trends, and impacts (contextualization).
3. Presentation on study developed by Aldunce et al. (2014b).
4. Individual questionnaire of attributes (second instrument): each participant chose 10 of 33 attributes identified by Aldunce et al. (2014b). This questionnaire aimed to identify the individual preferences of attributes of climate change resilience.
5. Discussion groups (third instrument): three discussion groups, each with a facilitator, were established in each workshop. The discussion section was divided into three parts: (1) the impacts of drought in each region; (2) the attributes of climate change resilience in their regional context; and (3) the characteristics of attributes and the roles of actor for building resilience communities.
  This paper focuses in the two questionnaires and the second discussion (2), where after the completion of the individual questionnaire on attributes (second instrument), each participant, supported by graphic material, chose the five most important attributes in the drought context, where all participants could see these new selections. The aim was to determine how the individual preferences changed after a dialogue with other participants.
6. Plenary: each group explained its results.
7. Final questionnaire on resilience [first instrument part (b)]: each participant filled the same initial questionnaire; this allowed us to trace the learning gained by participants during the workshop.

Participants were actors dealing with drought in some way, according to their experience with the problem. For instance, actors were selected on the basis of their experience with drought, as people directly affected by it (i.e., experienced a direct or indirect losses as a result of drought), as planners, planning, and managing impacts of drought, or as scholars, studying the phenomenon. The identification of actors was carried out taking an account of the territorial and governance contexts. Actors included were from government agencies, the academic sector, civil society organisations, and the private sector (individual questionnaires were anonymous; however, participants were able to indicate what kind of actors they identify with).

The three workshops were carried out in June 2014, plus an open seminar developed in September 2014, where results were presented. The data analysis included a thematic analysis of resilience definitions (Boyatzis 1998), through a deconstruction of definitions given by participants in the first instrument, and a descriptive statistic of frequency (Mackey and Gass 2015), allowing us to rank occurrences of each response.

Eight main principles guided the work in order to ensure the suitable participation of all actors (see Table 3). They were the basis of the research design, of the invitation process and of the workshops in order to sensitise to the participants (facilitators were prepared for contemplating it). The differences between the scientific and local knowledge were a special concern, related to classification, identification, and conceptualization of natural phenomena, and the contribution of the local knowledge transmission in this kind of processes, where socio-ecological system are highlighted (Warburton and Martin 1999; Singh et al. 2012). For this reason, the discussion phase emphasised these differences of understanding, enriching the dialogue on what the attributes mean.

**Table 3:** Guiding principles of research Sources: LaVeaux and Christopher (2009); Moodie (2010); Warburton and Martin (1999); Singh et al. (2013a)

Principles	Authors
(a) Acknowledge the relevance of community experiences	LaVeaux and Christopher (2009)
(b) Recognise the importance of each type of actor in the process	
(c) Respect different understanding and description of phenomena	Warburton and Martin (1999) LaVeaux and Christopher (2009)
(d) Facilitate collaboration between participants all the process	Moodie (2010)
(e) Inform strengths, limitations and scope of the process in order to reduce false expectations	Singh et al. (2013a , b)
(f) Promote a mutual learning	Moodie (2010)
(g) Disseminate results and knowledge	Moodie (2010)
(h) “Interpret data within the cultural context”	LaVeaux and Christopher (2009), p. 7

Two main criteria were applied in the process: shared understanding and the need to recognise the existence of asymmetries of power. The first was addressed through plenaries at the end of each workshop where a joint discussion served as a way to validate that the knowledge co-produced was agreeable to the participants. The second was addressed through the work of facilitators who were trained for safeguarding respect among participant and to include all points of views. Facilitators followed the same protocol and guide.

## Results and discussion: understanding local and national contexts

In this section, we present the results of the study and the discussion of the most relevant results and their implications for bringing resilience theory into practice. The section is divided into four parts: (1) distribution of participants by type of actors and territorial scale; (2) deconstruction of resilience; (3) attributes of resilience; and (4) general analysis.

### Distribution of participants

**Fig. 2:**
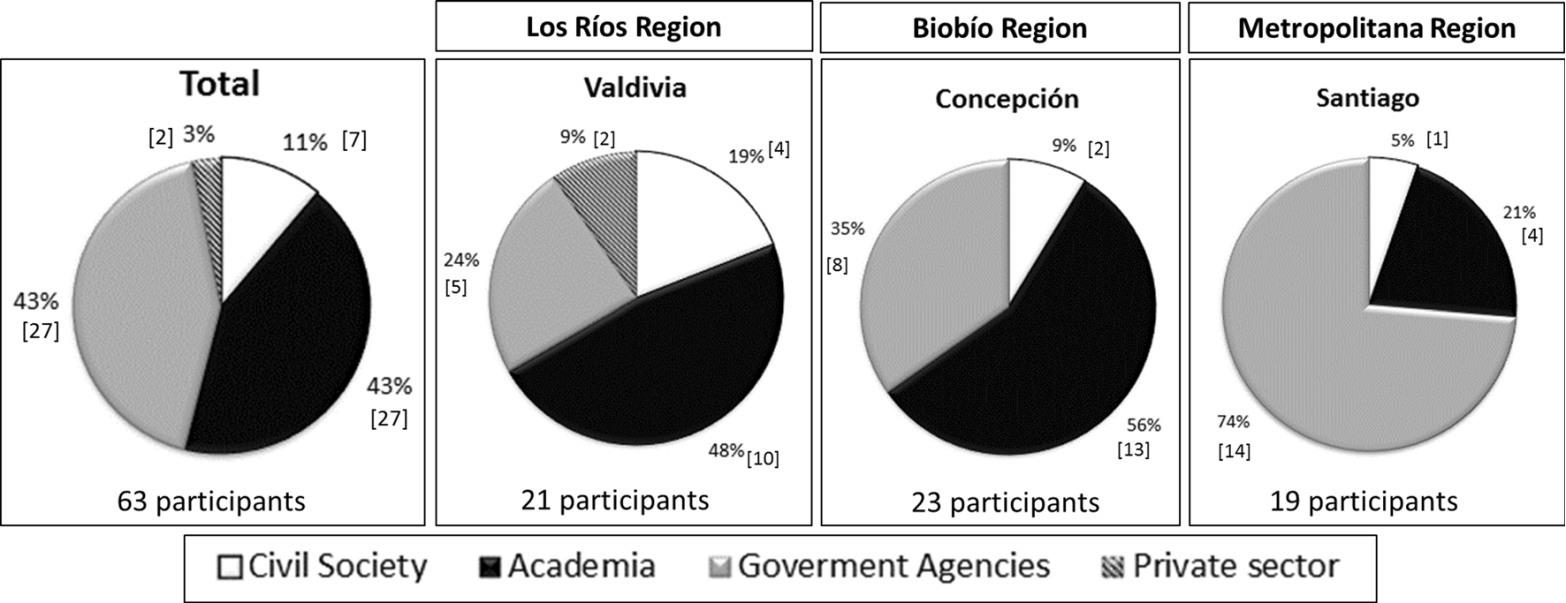
Total participants in workshops and by region

The academic sector and government agencies represent over 80 % of participants (Fig. 2). In Biobío and Metropolitan regions, there is no participation by the private sector, and civil society participation was less than one-tenth of participants. This was despite the broad invitation made to participate in the workshops, including a total of 289 invitations. The private sector had a participation rate of 6.7 %. In contrast, the academic sector had the highest participation rate (42.9 %). The total participation rate was 21.8 %.

The absence of private sector actors can be partially explained, because the perceptions of managers are that these kinds of processes require “expensive time investment with uncertain returns and at worst a risk to their perceived autonomy and independence” (Cash et al. 2003, p. 8090). On the other hand, the low level of participation from civil society can be due to a lack of “territorialisation” of policies on climate change in Latin America, a process in which a global phenomenon, such as climate change, is involved in local/regional territories and linked with local policies (Blanco and Fuenzalida 2013). In the Chilean case, a lack of territorialisation of policies on climate change is observed (Blanco and Fuenzalida 2013).

**Fig. 3:**
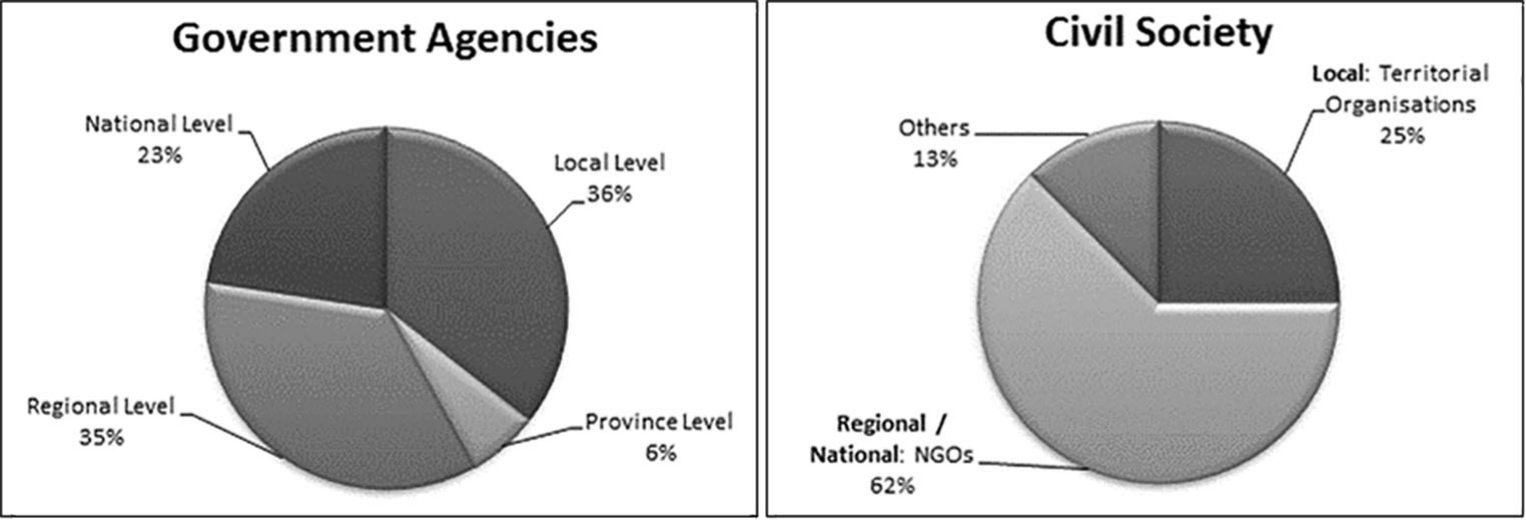
Distribution of government agencies and civil by territorial scale

Related to territorial scale (Fig. 3), the distribution of government agencies was balanced between local, regional, and national levels. The low presence of provincial actors is explained by the fact that in Chile, the provincial government is an administrative level with less representation than national and municipal levels. Civil society was comprised of NGOs and local organisations. The presence of more NGOs than local organisations can be explained by the fact that the NGOs are formal institutions, with clear direction, projects, leadership, and employees. In contrast, local organisations are not necessarily formally constituted and participation is voluntary. This difference, alongside the low degree of territorialisation of policies reflecting local conditions, can influence the level of participation of local organisations. In the case of the academic sector in Chile, universities have regional representation. It was not possible to sub-divide the private sector into types of participants due to the low level of participation.

### Deconstruction of resilience

**Fig. 4:**
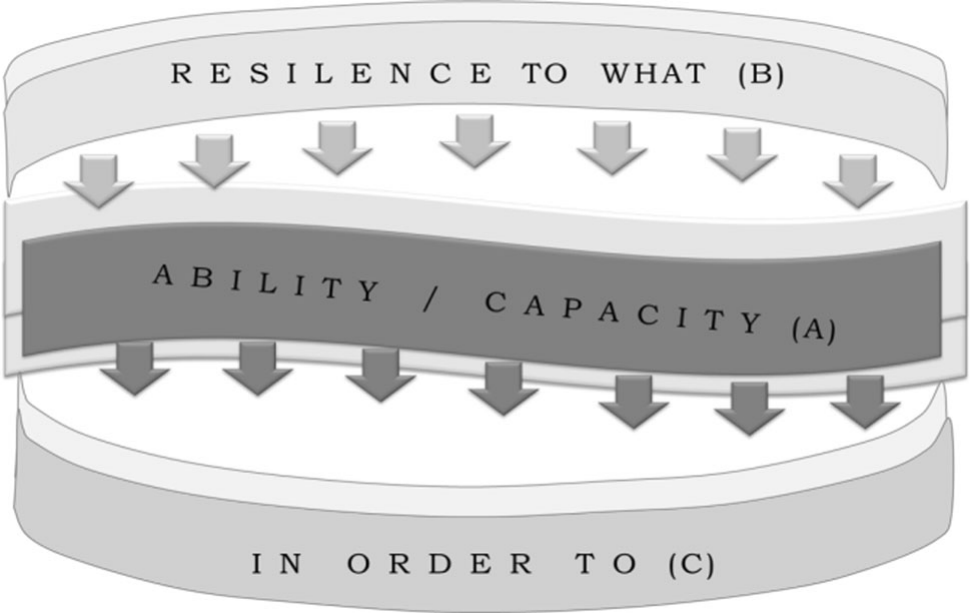
Deconstruction of resilience definition Source: (Aldunce et al. 2014b)

The first instrument (questionnaire on resilience) asked ‘what is resilience?’ at the beginning and the end of each workshop. A vast majority (95.5 %) of respondents understood resilience as an ability or capacity. Answers were deconstructed into three parts: ability or capacity for (A) whom, meaning the identity that is resilient; resilience to what? (B); and in order to achieve something (C) (see Fig. 4), following analysis realised by Aldunce et al. (2014b). Table 4 shows responses, divided by region and by topic according to the definition given by each participant (the order of appearance is according to the most named).

**Table 4:** Initial and final questionnaires

Region	Whom?		Ability/capacity (A)		To what (B)		In order to (C)	
	Initial	Final	Initial	Final	Initial	Final	Initial	Final
Los Ríos	No mentions System Ecosystem Socio-Ecosystem Individual/organism Social System	No mentions System Socio-Ecosystem	To recover To adapt To resist	To adapt To respond	Disturbance Adverse events	Adverse events	To return to an initial state	To maintain functions
Biobío	No mentions System Individual/organism Social System Ecosystem Socio-Ecosystem	No mentions Socio-Ecosystem System Individual/organism	To recover To resist	To adapt To recover	Disturbance Adverse events	Adverse events Change	To return to an initial state	To endure
Metropolitan	No mentions System Social System Ecosystem Socio-Ecosystem Individual/organism	No mentions System Socio-Ecosystem Social System Individual/organism	To recover To adapt To resist	To adapt To respond	Adverse events Change	Change	To return to an initial state To maintain essential characteristics	To maintain functions
Total	System Social System Socio-Ecosystem/Ecosystem	Socio-Ecosystem System	To recover To adapt To resist	To adapt To recover To respond	Disturbance Adverse events Change	Adverse events Change	To return to an initial state	To maintain functions To endure

In the initial questionnaire, half of the participants mentioned who is resilient: 39.4 % indicated that a system is resilient and 8.6 % indicated that individuals or organisms are resilient. Related to ability or capacity for ‘A’, there are no apparent important differences between regions. ‘To recover’ seems to be the most important verb associated with resilience (almost 50 % of mentions), followed by ‘to adapt’ (20.7 %) and ‘to resist’ (12.6 %). This is consistent with responses related to ‘in order to’ (C), where ‘to return to an initial state’ obtained 60 % of responses. The ideas about ‘the capacity to recover’ (A) and ‘to return to an initial state’ (C) were strongest in Biobío, where the former obtained a 74.1 % and the latter obtained a 100 % of mentions. In Biobío, these results could have been influenced by the earthquake in 2010 (Mw = 8.8), where this region was epicentre, with major losses and destruction.

The general idea of resilience given by participants refers to the ‘capacity’ of a ‘system’ ‘to recover’ from ‘disturbance’ in order ‘to return to an initial state’. It is interesting to observe that participants used verbs, such as ‘to recover’ and ‘to return’, concepts used mainly by disciplines in psychology and psychiatry to refer to individuals or human communities (Aldunce et al. 2014a).

Analysing by type of actor, it is interesting that definitions of public sector, civil society, and private sector included positive framing of resilience as the capacity for ‘recovering’ and ‘adapting’, and also ‘to learn’, ‘to transform’, and ‘to re-organise’, in the face of ‘adverse contexts’, ‘change’, or ‘new context’, where this new context is not necessarily negative. In contrast, the academic sector indicated only limited ideas of resilience as the capacity for ‘recovering’ to ‘disturbance’, ‘change’ and ‘adverse context’, associated mainly with negative expressions. Perhaps, this situation can be explained by the gap between science and society in Chile, according to Aldunce et al. (2014b), the inter-linkages between science and other actors have not been sufficiently fluid.

The same question was answered at the end of workshops. In the case of who is resilient, although ‘no mentions’ remained high, the ‘socio-ecological system’ emerged notably (64.3 % of participants who included this topic). In the same vein, other concept emerged, passing of recovering towards adapting, and to return to an initial state towards maintaining functions. Related to ‘resilience to what’, responses are very similar to the initial questionnaire: adverse events and change, but new responses included climate change, absent in the first questionnaire. In addition, the diversity of response decreased in the final questionnaire.

This shows that results were influenced by participation in the workshops and presentations given. This should be understood as a process of learning, implicating a change in who learns (van de Kerkhof and Wieczorek 2005), and a shared/distributed understanding/cognition of meaning in concepts and terminology (Pahl-Wostl et al. 2007). Even, as Olwig (2012), p. 118 argues, “local resilience is thus the result of local and global imaginaries”, where there is almost always an influence, which is necessary to recognise. An examination of this issue is analysed in the ‘‘General analysis’’ section.

### Attributes of resilience

**Fig. 5:**
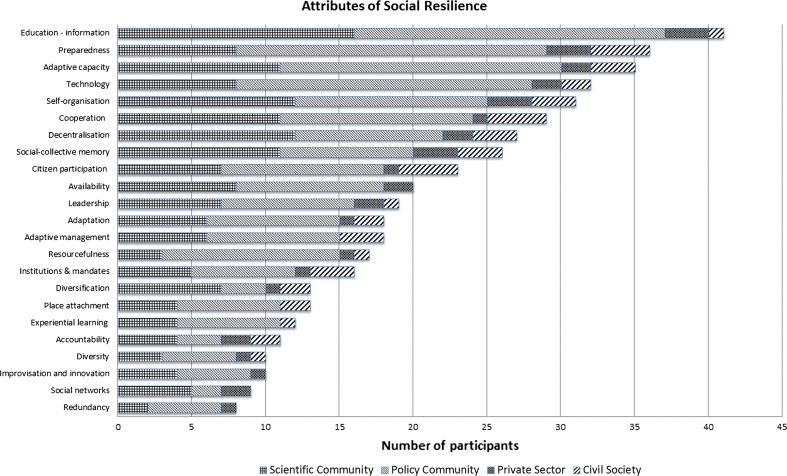
Score of attributes of social resilience

In this questionnaire, each participant could choose 10 attributes which they considered important for resilience building to drought in their contexts. This selection considered attributes of social and ecological resilience, but this paper focus on social resilience. Figure 5 shows the selection of attributes by type of actors.

‘Education-information’, the most voted attribute, refers to “opportune, equitable and universal access to information and education” (Aldunce et al. 2014b, p. 18). It is necessary to consider two aspects related to education. First, this topic has emerged as a main attribute in other studies, for example, Olwig (2012) and (Aldunce et al. 2015), who studied local understandings of resilience in Ghana and Australia, respectively. Second, it is necessary to understand recent Chilean history. The major student movements since the return of democracy were developed in 2006 and 2011, changing the policy agenda and generating a massive demand for public education (Fleet 2011; Cabalin 2012). The influence of this movement on citizens was relevant: for example, according to national polls, 80 % of the Chilean population supported the student movement (Cabalin 2012).

Preparedness was the second most mentioned attribute. Chile is exposed to a range of socio-natural and natural disasters, such as volcanic activity, earthquakes, tsunamis, floods, mudslides, and drought. Although earthquakes are not related to climate change, when participatory methodologies are developed, it is impossible to separate clearly different events, and disaster reduction policies in general embraces an all hazards approach. During the last earthquake and tsunami in Chile in 2010, preparedness was perceived as weak, generating a wide public discussion, which partially explains how this factor appeared relevant in workshops in Biobio and Metropolitan regions (earthquake covered the entire study area, but more intensely between Biobio and Metropolitan regions). Several studies have analysed the psychological impact of this earthquake (Gaborit 2010; Cova and Rincón 2010) and others have studied the slow action of the state (Acevedo 2013; Romero 2014). Uncertainty is a factor that affects psychologically due to lack of knowledge, while preparedness seeks to reduce this uncertainty (Naranjo Álvarez 2010; Mosquera and Gómez 2011).

**Fig. 6:**
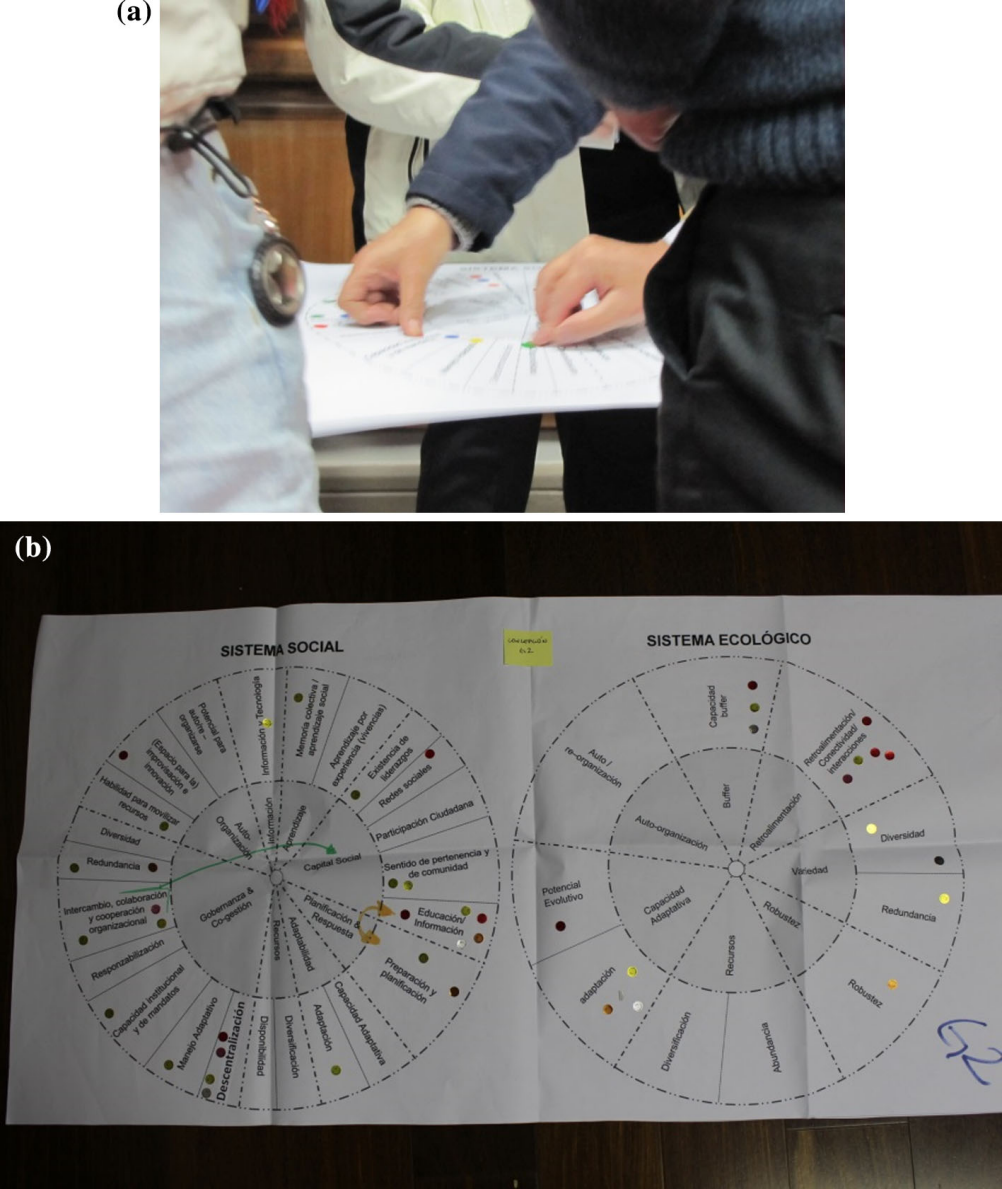
*Photographs* of the selection of attribute on ‘resilience wheel’ in workshops (**a**, **b**)

The second part of the attributes’ selection process was selecting the five most important attributes. This was done with the support of graphical representation afforded by the ‘Resilience Wheel’ by Aldunce et al. (2014b) (See Fig. 6), where all participants could see each other’s selections and discuss their choices. Table 5 ranks selections made on the questionnaire and the wheel, in total and by region. In this table, it is possible to see the diversity of preferences between the first (individual) and second (with discussion) selection during workshops.

**Table 5:** Attributes of social resilience: comparative ranking between individual and selection in group

	Total		Valdivia	
	Questionnaire	Wheel	Questionnaire	Wheel
1	Education-information	Education-information	Education-information	Education-information
2	Preparedness	Preparedness	Adaptive capacity	Technology
3	Adaptive capacity	Technology Cooperation	Self-organisation	Preparedness Cooperation Adaptive capacity
4	Technology	Decentralisation Citizen participation	Decentralisation	Political will

	Concepcion		Santiago	
	Questionnaire	Wheel	Questionnaire	Wheel
1	Education-information	Education-information	Preparedness	Education-information Preparedness
2	Preparedness	Place attachment	Adaptive capacity	Technology
	Decentralisation	Decentralisation	Technology	Cooperation
3	Technology Self-organisation	Citizen participation	Education-information	Institutions and mandates
4	Leadership Social-collective memory Cooperation	Leadership Preparedness Accountability	Cooperation Institutions & mandates	Leadership Citizen participation Place attachment Adaptive management

There are no apparent differences in the selections of the most important attribute, where ‘Education-information’ emerged as the most substantial aspect. Perhaps, Metropolitan region is highlighted, because initially participants considered ‘Education-Information’ in third place, however, in the second selection, this attribute was considered as first. This change can be partly explained by the fact that in this region, 73.7 % of participants were from public sector, whose field of work is in planning and management. Therefore, the questionnaire collected individual preferences, and in general, these are associated with own ideas according to the performance area. However, in the second selection, participants dialogued with other actors about their choices, reaching the same pattern as in the other regions. As van de Kerkhof and Wieczorek (2005) state, the transitional process of mutual learning requires that actors leave their own interests and ideas, opening their mind to other perspectives and information. Therefore, the group discussion allowed this transition between individual viewpoints to integrated ideas. This process can explain the importance of the attributes: ‘cooperation’ and ‘citizen participation’, during the second selection of attributes.

It is interesting to note the emergence of ‘decentralisation’ and the inclusion of ‘political will’. With respect to decentralisation, Chile is a centralised country where the Metropolitan region is the political power, home to the leading national institutions, and where most important decisions are made. This situation has been claimed historically by citizen and politicians of other regions. Although there has been an effort to decentralise undertaken by the central government, for example, the creation of Los Ríos region in 2007, this effort is perceived as weak. Regions continue to demand more independence, considering this factor as determinant of their limited development (Montecinos 2005; Waissbluth et al. 2007). For this reason, it is not strange that this aspect will be important in Los Ríos and Biobío, but absent in the Metropolitan region.

Regarding ‘political will’, its inclusion in the Los Ríos workshop can be associated with ‘decentralisation’ and the perception of a lack of political will, in order to, achieve it. According to Waissbluth et al. (2007), Chilean political elites have historically considered that regions and municipalities do not have the capacity for managing greater responsibilities. Tagle (1985) argues that Chilean institutions are monopolised by politicians from the capital to the detriment of regional and local interests. This aspect allows us to connect the emergence of decentralisation and political will with the lack of territorialisation that, at the same time, could partially explain the lower participation rate of private sector and civil society.

‘Preparedness’, ‘education-information’, ‘decentralisation’, and ‘political will’, among others, have important implications on governance, because these concepts are closely linked to proactivity, innovation, adaptation, awareness, warning, self-management, and training, promoting local responsibility and a more balanced decision-making (Aldunce et al. 2015). Therefore, they involve moving from the response or reactive resilience to proactive resilience, which some scholars argues is where societies should be moving towards (Aldunce et al. 2015; Handmer and Dovers 1996).

### General analysis

Overall, there is no marked difference between regions, where issues of national interest, such as ‘education-information’ and ‘preparedness’, are highlighted over others. Given these results, it seems important to note the type of actor more so than the place where they live into a national context. This reinforces why special attention must be given to the different understandings in knowledge co-production processes. However, regional and local aspects considered historically relevant, such as ‘decentralisation’ or ‘political will’, emerged as differentiators. In addition, to be involved in a discussion process, knowledge and mutual learning seem to have a high influence, generating a similar distribution of final responses and decreasing their diversity. This point should not be seen as negative, but as a normal process of learning. Indeed, according to van de Kerkhof and Wieczorek (2005) and Pahl-Wostl et al. (2007), any kind of learning implies a change in actions or knowledge of who learns, capturing the core of social learning.

In the case study, where it was necessary to identify the understandings of resilience and its attributes among participants, the learning process led to consensus. However, caution should be exercised, because in other cases, it is deliberation rather than consensus that is desired, ideally improving the long-term contribution of stakeholders in policy processes (van de Kerkhof 2006).

The ethical dimension was fundamental in order to organise and perform our research. Principles included in Table 3 were the basis of this research, because our concern was to avoid false expectations (Singh et al. 2013a), respect different understandings (Warburton and Martin 1999), balance power relations between participants, and avoid treating communities as data extraction sources, called by Moodie (2010) as “helicopter research”. In our view, not only do these aspects allow for trust in the research team, which is fundamental for achieving involvement and engagement, but also they must be considered the core of researchers’ behaviour. In particular, power asymmetries were faced through individual questionnaires and discussion groups. The former allows us to have responses with the same weight/influence on the result. For discussion groups, recognising that power asymmetries cannot be fully placated, the role of facilitators was crucial. They were trained to face with it.

As Miller et al. (2010), p. 16 emphasise, “resilience research can help to design opportunities for reflection and learning, and appropriate networks, institutions, and governance structures”. In this context, the diversity of meanings and understanding of resilience in the literature should be seen as an opportunity for the co-production of knowledge, because it allows us to build contextualised attributes, and their measures and strategies of application. Involving stakeholders in the design and decision process that frames their own development, generating the necessary engagement and empowerment in order to build and sustain long-term policy implementation (Wolf and Moser 2011).

In this study, we show how resilience theory can be articulated into practice, addressing 4 of the 5 “dimensions of knowledge co-production” raised by Armitage et al. (2011), p. 999: knowledge gathering through questionnaires; knowledge sharing through discussion groups; knowledge integration through discussion groups and plenary; and knowledge interpretation through the analysis and the discussion during the seminar of return results. In order to incorporate the knowledge application (fifth dimension), we consider that efforts for co-producing knowledge are linked to political decisions. By doing so, they have the potential first to institutionalise the process, giving support, continuity, and financing; second, it would approach territorial realities; and third, it would encourage participation and empowerment in different levels of governance.

Decentralisation as a political process and territorialisation as a socio-natural process seem to be key points in the development of this kind of work in order to move not only from theory to practice, but also towards real applicability, because the current context discourages the participation of local actors beyond public and academic sectors. Therefore, one of the challenges is to create bridges that increase motivation for participating.

The participation of scientists in the process was important. Regarding this participation, Blanco and Fuenzalida (2013) explain, one challenge for academia is to understand that we are not neutral entities generating pure information, supplemented by Cornell et al. (2013), p. 62 who argue that “it also requires an awareness and willingness on the part of the science community to accept this responsibility for transformation and engagement, while acknowledging the contested and political nature of responding to global change.” The workshops conducted in this study gave a real opportunity for these scientists to engage with other actors in the process of co-producing knowledge.

## Conclusions

Motivated by the clear need for concrete and demonstrable means of bringing theory to practice on issues of resilience through participatory processes, the research process in this study included: first, to take a systematic review on resilience to climate change; second, to carry out an actor search with an inclusive methodology that gathers up visions and perception through individual and discussion processes in order to analyse the theory; third, quantitative and qualitative instruments and ludic tools of works; fourth, to create the participation environment in workshops through training of facilitators; and finally, to analyse and generate a devolution process.

At least four lessons related to methods and approaches emerge from this knowledge co-production:

- This research demonstrates that theory can be articulated into practice through different instruments and tools if knowledge dimensions are considered.
- Principles and ethical dimension should be included from the design phase onward.
- The facilitator role is crucial, and therefore, protocol, guide of work, and training are fundamental.
- In the same vein that Armitage et al. (2011) identify, how to face differences in understanding, shared understanding, power relations, and normative context seems to be important challenges for the co-production of knowledge.

The applicability of this kind of process in other places can be achieved, but any process needs to be contextualised; therefore, first, the research team must know historical, social, environmental, political, and economic realities; second, language, tools, and provided information must allow participants to understand the process. If this aspect is not achieved; asymmetries are increased during the process.

From emerged results, key lessons can be considered for policy makers and society. First, when efforts for building resilience are made, understanding resilience as an ability or capacity gives the opportunity to focus on socio-ecological aspects. Second, the framing of resilience as a capacity not only ‘to recovering’, but also ‘to adapt’, ‘to learn’, ‘to transform’, and ‘to re-organise’, opens up opportunities to move towards continually improved systems. Third, equitable/universal access to information and education and to enhance preparedness emerged as key for building resilience, especially in countries frequently exposed to disasters related to climatic hazards.

Resilience and its diversity in meanings and understanding gave us an opportunity for co-producing knowledge, fulfilling the demand for the opening up of knowledge systems, with an intensification of relationship and collaboration between science and other actors, and an academic practice more oriented toward society. We find that it is relevant and pertinent to develop co-production processes in the context of resilience to climate change, because at least it allows us to communicate resilience from contextualised realities and languages, facilitating the engagement and trust of stakeholders, and establishing the legitimacy of the process.

However, based on our experience in this study, it is important to understand that this kind of effort is best served through permanent or more sustained efforts in collaboration with society. Furthermore, we consider that an explicit link to political decisions and context could facilitate institutionalised continuity and financial prioritisation; however, this remains to be affirmed through follow-up studies on ex-post policy evaluations. In our case, contextualisation to local realities could also empower processes to facilitate more meaningful decentralisation of decisions as a political process. In the Chilean context, these seem to be key points of consideration in the development of this kind of work. Many challenges remain, including how to generate regular dialogue and engagement between science, government, and society, where scientists assume part of the responsibility for transformation and engagement as an actor (at the same level of the others), making their own research open to public scrutiny, and to develop transdisciplinary work in order to enrich the research process and contextualise it.

## Footnotes

1 Working paper available in: http://www.cr2.cl/wp-content/uploads/2016/06/Aldunce-et-al-2014-Working-paper-Conceptualization-Resilience.pdf.
